# American Medical Association and Medical Education

**Published:** 1857-09

**Authors:** 


### American Medical Association and Medical Education.

We have already said so much upon this subject that it has become wearisome, even to us, and we fear much more so to our readers, and yet we hope they will pardon us for a few additional remarks, suggested by some of the singular positions, assumed in reference to this matter.

The New Orleans Medical News and Hospital Gazette asks, "Is this Association is a representation of the medical schools of the land; or is it a National Congress of the "people," of the profession?" and says, in *partial* reply to its own question, "no man will deny that it *purports* to be the latter," and there lies the difficulty, which we suggested in the last number of this Journal; it *purports* to be what it is not in reality, and, as suggested upon a former occasion, cannot in the very nature of things become. The "people" of the profession, we say, never have been represented, and while they should be, to accomplish the objects which are supposed by some to belong legitimately to it, the notorious fact is, that it has been controlled by a comparatively few of the profession, some of the most prominent of whom are connected with Medical Colleges, and have not hesitated to use it for their own advancement, and have yet hope of making it minister still farther to the accomplishment of their selfish designs.

The truth upon this subject is, that the mass of the profession have never been aroused to an interest in this body, and even if its workings and results were entirely unobjectionable, the vast country and varying circumstances, embraced in the area, over which it is proposed that it should exercise "control," would render the attempt utterly futile. You cannot get the "*people*" of the profession to send representatives, and its management is left to a few prominent, and, in many instances, self-important members of the profession, who occupy the more conspicuous positions.

We know whereof we speak, from an observation of the practical workings of this body; we *know* that instrumentalities for the accomplishment of certain ends not entirely creditable, even to political bodies, are brought into operation, the details of which are well calculated to disgust those who have really looked to the Association for the advancement of science, rather than of individuals, and furnish a sufficient explanation of the meagre attendance at the last meeting.

We go still further, and assert it as our belief, that so far from the controlling influence in the association being of a *popular* character, (in a professional sense of course,) it has belonged to the Colleges, and was most strikingly manifested at the last meeting of the Association, and desiring, as we do, most earnestly, the establishment of the body upon a permanent basis, we go in heartily for the exclusion of the Schools from representation, and agree with the sentiment that "the representative of a medical society can scarcely feel comfortable in his seat when he reflects that the highest honors of the Association is each year sure to be conferred on one whom he believes to be sapping the foundation of the Association, and that a School of seven men is entitled to a more numerous relative representation, by far, than even the State Medical Society, from which he may bring his credentials; much less can he even dream of "advancement," "harmony" and "the matter of reports." This is all our doctrine and an approval of our position—that as at present constituted, the American Medical Association *cannot and should not* "*control*" the American Medical *profession.*

We are and expect to be a supporter of this body, for legitimate and proper ends, but in reference to this, as other organizations of a *so-called National character*, we must be permitted to exercise great caution. The day is indeed past, when we were willing to commit our rights, whether political, religious, or of other kinds, into the hands of National organizations.

We are an advocate, however, for the elevation of the standard of Medical education, and shall give our feeble support to any means calculated, in our judgment, or in the judgment of the profession generally, to effect the end.

We shall, however, continue to protest against the use of the American Medical Association, as an instrument, in the hands of any College or set of Colleges, for the purpose of bringing others into disrepute, which in plain terms, we consider to be the sum and substance of the present movement upon this subject. The truth is, the simple and practical plan, under existing circumstances, is to leave the whole question to the Colleges and the "masses" of the profession. Let each institution be brought into a competition for offering the best advantages, and let these dear "people" of the profession settle the question of merit, by their support or otherwise—this is the plain common sense way of settling the whole question.

